# Early mepolizumab initiation during remission induction is associated with lower organ damage burden in EGPA: a multicentre cohort study

**DOI:** 10.1093/rheumatology/keag523

**Published:** 2026-09-21

**Authors:** Akihito Maruyama, Nobuyuki Ono, Tatsuya Kai, Yasutaka Kimoto, Miki Nakano, Yasushi Inoue, Naoyasu Ueda, Tomoya Miyamura, Kyoko Otsuka, Yuya Shinkawa, Yoshifumi Tada, Mitsuteru Akahoshi, Hiroaki Niiro

**Affiliations:** Department of Rheumatology, Faculty of Medicine, Saga University, Saga, Japan; Department of Rheumatology, Fukuoka City Hospital, Fukuoka, Japan; Department of Clinical Immunology, Rheumatology, and Infectious Disease, Kyushu University Hospital, Fukuoka, Japan; Department of Medicine and Biosystemic Science, Kyushu University Hospital, Fukuoka, Japan; Department of Rheumatology, Fukuoka Red Cross Hospital, Fukuoka, Japan; Department of Rheumatology, Fukuoka Red Cross Hospital, Fukuoka, Japan; Department of internal medicine, Miyazaki Prefectural Miyazaki Hospital, Miyazaki, Japan; Department of Rheumatology, National Hospital Organization Kyushu Medical Center, Fukuoka, Japan; Department of Rheumatology, Kyushu Railway Memorial Hospital, Kitakyushu, Japan; Clinical Research Center, Saga University Hospital, Saga, Japan; Department of Rheumatology, Faculty of Medicine, Saga University, Saga, Japan; Department of Rheumatology, Faculty of Medicine, Saga University, Saga, Japan; Department of Medical Education, Graduate School of Medical Sciences, Kyushu University, Fukuoka, Japan

**Keywords:** eosinophilic granulomatosis with polyangiitis, mepolizumab, remission induction, organ damage, anti-IL-5

## Abstract

**Objective:**

The objective of this study was to evaluate whether early mepolizumab initiation during remission induction is associated with favourable clinical outcomes and lower organ damage burden in patients with eosinophilic granulomatosis with polyangiitis (EGPA).

**Methods:**

This retrospective multicentre cohort study used data from the Kyushu Vasculitis Cohort Study. Patients with EGPA were classified into an early mepolizumab group, in which mepolizumab was initiated within 3 months of remission induction, and a conventional group. The primary end point was clinical remission at 1 year, defined as BVAS = 0 with prednisolone ≤4 mg/day. Secondary end points included remission at 6 months and 2 years, glucocorticoid exposure, relapse, mortality, and organ damage as assessed by the Vasculitis Damage Index (VDI). Longitudinal VDI values from 6 months to 2 years were analysed using a linear mixed-effects model.

**Results:**

Sixty-four patients were included (early mepolizumab, *n* = 16; conventional, *n* = 48). Clinical remission at 1 year was achieved more frequently in the early mepolizumab group than in the conventional group (69% *vs* 27%; *P = *0.006). At 2 years, remission was achieved in 10/10 (100%) *vs* 13/45 (29%) evaluable patients (*P < *0.001), and glucocorticoid discontinuation was achieved in 4/10 (40%) *vs* 4/45 (9%) of those patients (*P = *0.029). BVAS decreased markedly in both groups. Early mepolizumab initiation was associated with lower longitudinal VDI values (β = −0.36, 95% CI −0.70 to −0.03; *P = *0.032).

**Conclusion:**

Early mepolizumab initiation was associated with higher remission rates, lower glucocorticoid exposure and lower organ damage burden during the first 2 years in EGPA.

*Rheumatology* key messages

- Early mepolizumab initiation during remission induction was associated with lower organ damage burden in EGPA.
- Early initiation was associated with higher 1-year remission and lower glucocorticoid exposure.
- Despite reduced vasculitis activity in both groups, established peripheral neuropathy frequently persisted as irreversible damage.

## Introduction

Eosinophilic granulomatosis with polyangiitis (EGPA) is a rare systemic necrotizing vasculitis characterized by asthma, peripheral eosinophilia, and small-vessel inflammation leading to multi-organ involvement [1]. EGPA is classified as an ANCA-associated vasculitis according to the Chapel Hill Consensus Conference nomenclature and presents with heterogeneous clinical manifestations affecting the peripheral nervous system, lungs, skin, and cardiovascular system [2]. Although survival has improved substantially with modern therapy, relapses and irreversible organ damage remain important causes of mortality.

Glucocorticoids (GCs) are the cornerstone of remission induction therapy in EGPA, often combined with immunosuppressive agents such as CYC in severe disease [3]. Although effective, relapses frequently occur during GC tapering [4], and treatment-related toxicity remains an important concern with prolonged GC exposure [3] Conventional immunosuppressive agents also cause significant toxicity, including infection risk and treatment-related complications [5]. Therefore, treatment strategies that achieve effective disease control while reducing GC exposure and organ damage are needed.

IL-5 plays a central role in EGPA pathogenesis by promoting eosinophil proliferation, activation, and survival. Mepolizumab, a monoclonal antibody targeting IL-5, has demonstrated efficacy in EGPA [6, 7]. The MIRRA randomized controlled trial showed that mepolizumab significantly increased remission duration and reduced GC exposure compared with placebo in patients with relapsing or refractory EGPA [8]. Long-term extension studies further confirmed sustained remission and continued GC reduction, with GC discontinuation achieved in ∼28% of patients [9]. The MARS study demonstrated long-term safety and effectiveness of mepolizumab in Japanese patients with EGPA, with GC discontinuation achieved in 36% of patients at 96 weeks [10]. These findings establish mepolizumab as an effective therapy for disease control and GC sparing in EGPA.

Real-world observational studies have further supported improved disease control and reduced GC exposure with mepolizumab in routine clinical practice [11–16]. However, most previous studies have focused on relapsing or refractory disease, and the potential benefit of earlier mepolizumab initiation during remission induction remains incompletely understood.

Irreversible organ damage is a major determinant of long-term prognosis in vasculitis and is commonly assessed using the Vasculitis Damage Index (VDI), a validated instrument for measuring cumulative organ damage [17, 18]. Organ damage in EGPA may result from both persistent disease activity and treatment-related toxicity, particularly prolonged GC exposure. Therefore, early and effective disease control combined with reduction in GC exposure may help prevent organ damage accrual and improve long-term outcomes. To our knowledge, few multicentre real-world studies have examined whether early mepolizumab initiation during remission induction is associated with subsequent organ damage accrual in EGPA. The Kyushu Vasculitis Cohort (KVAS) is a multicentre observational cohort of ANCA-associated vasculitis that provides longitudinal real-world data on treatment and outcomes [19, 20]. Using this cohort, we investigated whether early initiation of mepolizumab during remission induction therapy is associated with favourable clinical outcomes and lower longitudinal VDI values during the first 2 years compared with conventional remission induction therapy in patients with EGPA.

## Methods

### Study design and cohort

This retrospective multicentre cohort study was conducted using data from KVAS, an ongoing observational cohort established in April 2010 across 14 institutions in Japan. KVAS includes patients diagnosed with ANCA-associated vasculitis, including microscopic polyangiitis, granulomatosis with polyangiitis, and EGPA, and its design and characteristics have been described previously [19, 20].

Patients diagnosed with EGPA between April 2011 and July 2024 and registered in KVAS were eligible for inclusion. EGPA was classified according to the European Medicines Agency algorithm and fulfilled the 2022 ACR/EULAR classification criteria [21, 22].

Patients who initiated mepolizumab within 3 months of the start of remission induction were defined as the early mepolizumab group. Patients who did not receive mepolizumab within this 3-month period were defined as the conventional group. Patients who initiated mepolizumab after 3 months were included in the conventional group. All patients treated with mepolizumab received 300 mg subcutaneously every 4 weeks, the approved dosing regimen for EGPA in Japan. The timing of mepolizumab initiation was determined by the treating physician. No standardized protocol was applied across the participating centres regarding the timing of mepolizumab initiation, GC tapering, or the initiation, continuation, tapering, or discontinuation of conventional immunosuppressive agents. These treatment decisions were individualized according to disease activity, organ involvement, treatment response, and treatment tolerability. The specific patient-level reasons for early or delayed mepolizumab initiation were not systematically recorded in the cohort database.

### Clinical assessment and data collection

Clinical data were collected retrospectively from medical records. Baseline was defined as the time of initiation of remission-induction therapy for EGPA, rather than the time of mepolizumab initiation. Baseline variables included age, sex, disease duration, ANCA status, and treatment regimens.

Disease activity was assessed using the BVAS, a validated instrument for measuring disease activity in systemic vasculitis [23, 24]. Organ damage was assessed using the VDI, which quantifies irreversible organ damage resulting from vasculitis or its treatment [17, 18]. Clinical remission, prednisolone dose, and VDI were evaluated at predefined time points of 6 months, 1 year and 2 years after the start of remission induction. Relapses and adverse events were assessed during the predefined intervals of 0–12 months and 12–24 months. Relative change in PSL dose was calculated as (PSL dose at each follow-up time point / maximum PSL dose during remission induction) − 1.

### Definitions

Clinical remission was defined as BVAS = 0 and prednisolone ≤4 mg/day at each predefined assessment time point. [8]. Relapse was defined as recurrence of vasculitis requiring treatment escalation, including either an increase in GC dose of ≥10 mg/day, or initiation or escalation of immunosuppressive therapy. Organ damage burden was assessed using the VDI at each predefined time point [25]. GC-related VDI was expressed as the sum of VDI items that we considered to be adverse effects due to GCs: muscle atrophy and weakness, osteoporosis with fracture or vertebral collapse, diabetes mellitus, cataracts, and avascular necrosis.

### Study end points

The primary end point was clinical remission at 1 year after remission induction therapy, defined as BVAS = 0 and prednisolone ≤4 mg/day.

Secondary end points included:

- Clinical remission at 6 months and 2 years
- Ideal remission (BVAS = 0 with complete GC discontinuation) at 1 year and 2 years
- Relapse during predefined intervals (0–12 months, and 12–24 months)
- Prednisolone dose at 6 months, 1 year, and 2 years
- Adverse events and severe infections during predefined intervals (0–12 months, and 12–24 months)
- Death during the observation period
- Organ damage assessed by VDI and GC-related VDI items at 6 months, 1 year and 2 years
- Longitudinal change in VDI over time.

### Statistical analysis

Continuous variables were presented as median (range), and categorical variables as number (percentage). Between-group comparisons were performed using the Wilcoxon rank-sum test for continuous variables and Fisher’s exact test for categorical variables.

No formal sample-size calculation was performed; the study size was determined by the number of eligible patients available in the KVAS database.

Longitudinal VDI values at 6 months, 1 year and 2 years were analysed using a linear mixed-effects model with treatment group and assessment time as fixed effects and a patient-specific random intercept. Assessment time was treated as a categorical variable, with 6 months as the reference time point. The treatment-group coefficient represented the average between-group difference in VDI across the three assessment time points. All available repeated measurements within the 2-year analysis window were included, and estimated marginal means were derived from the fitted model for graphical presentation. Analyses at each predefined time point were based on the available cases, and no imputation was performed. To assess potential calendar-time bias, a sensitivity analysis was performed among patients who underwent remission induction in or after 2019.

Statistical analyses were performed using JMP version 18 (SAS Institute, Cary, NC, USA) and R version 4.5.2 (R Foundation for Statistical Computing, Vienna, Austria) [26]. Linear mixed-effects models were fitted using the lme4 package, and statistical inference was performed using the lmerTest package [27, 28].

All statistical tests were two-sided, and *P* values < 0.05 were considered statistically significant.

## Results

### Patient characteristics

A total of 78 patients with EGPA were registered in the Kyushu Vasculitis Cohort during the study period. Among these, 65 patients had at least 1 year of follow-up after remission induction therapy. One patient was excluded due to missing data, and 64 patients were included in the final analysis. Of these, 16 patients (25%) were classified into the early mepolizumab group and 48 patients (75%) into the conventional group.

The baseline characteristics are summarized in Table 1. Except for older age at disease onset in the early mepolizumab group than in the conventional group (median, 67 *vs* 53 years; *P = *0.015), no significant between-group differences were observed in sex, history of asthma, baseline BVAS, or major organ involvement. MPO-ANCA positivity was observed in 7 patients (44%) in the early mepolizumab group and in 19 patients (40%) in the conventional group. The median observation period was significantly shorter in the early mepolizumab group than in the conventional group [24.9 months (range, 12.3–37.5) *vs* 37.3 months (range, 12.0–121.5); *P = *0.002). In the conventional group, eight patients initiated mepolizumab at >3 months but within 1 year after remission induction, and one additional patient initiated mepolizumab between the 1- and 2-year assessments. One patient discontinued mepolizumab during the latter interval; the exact timing and reasons for these treatment changes were unavailable. Conventional immunosuppressive agents were used more frequently in the conventional group. Within 6 months of remission induction, immunosuppressive agents were used in 35 patients (73%) in the conventional group and in 6 patients (38%) in the early mepolizumab group (*P = *0.016). At 1 year, the corresponding proportions were 52% and 13% (*P = *0.008). At 2 years, among patients with available data, conventional immunosuppressive agents were used in 29 of 45 patients (64%) in the conventional group and in 1 of 10 patients (10%) in the early mepolizumab group (*P = *0.003). The detailed distribution of individual immunosuppressive agents at 6 months is provided in Supplementary Table S1.

**Table 1:** Baseline characteristics of patients with EGPA in the conventional remission induction group and the early MPZ initiation group.

	All patients (*N* = 64)		Early MPZ group (*N* = 16)		Conventional group (*N* = 48)		*P* value
Male:female (female%)	27:37	(57.8)	6:10	(62.5)	21:27	(56.3)	0.774
Median age at diagnosis (range)	58	(29–88)	67	(31–88)	53	(29–84)	0.015*
Observation period, months (range)	36.0	(12.0–121.5)	24.9	(12.3–37.5)	37.3	(12.0–121.5)	0.002**
Bronchial asthma, *n* (%)	38	(59.4)	11	(68.8)	27	(56.3)	0.558
MPO-ANCA, *n* (%)	26	(40.6)	7	(43.8)	19	(39.6)	0.777
PR3-ANCA, *n* (%)	3	(4.7)	0	(0.0)	3	(6.3)	0.567
Median initial BVAS (range)	16	(3–32)	14	(6–32)	16	(3–30)	0.732
BVAS clinical symptoms (%)							
Systemic symptoms (%)	42	(65.6)	10	(62.5)	32	(66.7)	0.769
Neurological (%)	56	(87.5)	15	(93.8)	41	(85.4)	0.667
Skin (%)	32	(50.0)	8	(50.0)	24	(50.0)	1.000
Ophthalmic/otologic/rhinologic (%)	39	(60.9)	10	(62.5)	29	(60.4)	1.000
Pulmonary (%)	28	(43.8)	6	(37.5)	22	(45.8)	0.772
Cardiac (%)	14	(21.9)	6	(37.5)	8	(16.7)	0.095
Renal (%)	12	(18.8)	2	(12.5)	10	(20.8)	0.714
Gastrointestinal (%)	3	(4.7)	0	(0.0)	3	(6.3)	0.567
Treatment							
Median maximum PSL dose, mg (range)	50	(20–75)	50	(30–65)	50	(20–75)	0.312
Initial GC pulse, *n* (%)	30	(46.9)	7	(43.8)	23	(47.9)	1.000
Median PSL after 6 months, mg (range)	9	(1–40)	6	(1–13)	10	(3–40)	0.031*
Median PSL after 1 year, mg (range)	5	(0–50)	5	(0–11)	6.5	(0–50)	0.001**
Median PSL after 2 years, mg (range)	5	(0–15)	1.5	(0–4)	5	(0–15)	<0.001***
Immunosuppressant combination within 6 months, *n* (%)	41/64	(64.1)	6	(37.5)	35	(72.9)	0.016*
Immunosuppressant combination after 1 year, *n* (%)	27/64	(42.2)	2	(12.5)	25	(52.1)	0.008**
Immunosuppressant combination after 2 years, *n* (%)	30/55	(54.5)	1/10	(10.0)	29/45	(64.4)	0.003**
Prognosis							
BVAS = 0–defined remission, *n* (%)	62	(96.9)	16	(100.0)	46	(95.8)	1.000
Clinical remission achieved at 6 months, *n* (%)	10	(15.6)	5	(31.3)	5	(10.4)	0.104
Clinical remission achieved at 1 year, *n* (%)	24	(37.5)	11	(68.8)	13	(27.1)	0.006**
Clinical remission achieved at 2 years, *n* (%)	23/55	(41.8)	10/10	(100.0)	13/45	(28.9)	<0.001***
Ideal remission at 1 year, *n* (%)	4	(6.3)	2	(12.5)	2	(4.2)	0.258
Ideal remission at 2 years, *n* (%)	8/55	(14.5)	4/10	(40.0)	4/45	(8.9)	0.029*
Relapse within 1 year, *n* (%)	5	(7.8)	0	(0.0)	5	(10.4)	0.320
Relapse within 1–2 years, *n* (%)	3/55	(5.5)	0	(0.0)	3/45	(6.7)	1.000
Adverse events within 1 year, *n* (%)	12	(18.8)	1	(6.3)	11	(22.9)	0.266
Adverse events within 1–2 years, *n* (%)	7/55	(12.7)	1/10	(10.0)	6/45	(13.3)	1.000
Severe infections within 1 year, *n* (%)	6	(9.4)	1	(6.3)	5	(10.4)	1.000
Severe infections within 1–2 years, *n* (%)	3/55	(5.5)	0	(0.0)	3/45	(6.7)	1.000
Death during follow up, *n* (%)	1	(1.6)	0	(0.0)	1	(2.1)	1.000

Baseline demographic, clinical, and laboratory characteristics were assessed at the start of remission-induction therapy, rather than at the time of mepolizumab initiation. Treatment and outcome data were assessed at the indicated follow-up time points. Continuous variables are expressed as median (range), and categorical variables are expressed as number (percentage). Between-group comparisons were performed using the Wilcoxon rank-sum test for continuous variables and Fisher’s exact test for categorical variables. *P < 0.05, **P < 0.01, ***P < 0.001. Abbreviations: EGPA: eosinophilic granulomatosis with polyangiitis; MPZ: mepolizumab; VDI: Vasculitis Damage Index; GC: glucocorticoid; PSL: prednisolone.

### Changes in disease activity

BVAS decreased markedly in both groups, with no significant between-group differences at any time point (Fig. 1). Organ involvement according to the BVAS domains decreased markedly after remission induction in both groups (Supplementary Table S2). At 6 months and 1 year, no active BVAS domains were recorded in the early mepolizumab group. In the conventional group, neurological involvement was the most frequently recorded residual domain, occurring in 5 of 48 patients (10%) at 6 months and in 6 of 48 patients (13%) at 1 year. These residual neurological manifestations consisted mainly of sensory peripheral neuropathy and motor mononeuritis multiplex. At 2 years, no active neurological involvement was observed in either group.

**Figure 1:**
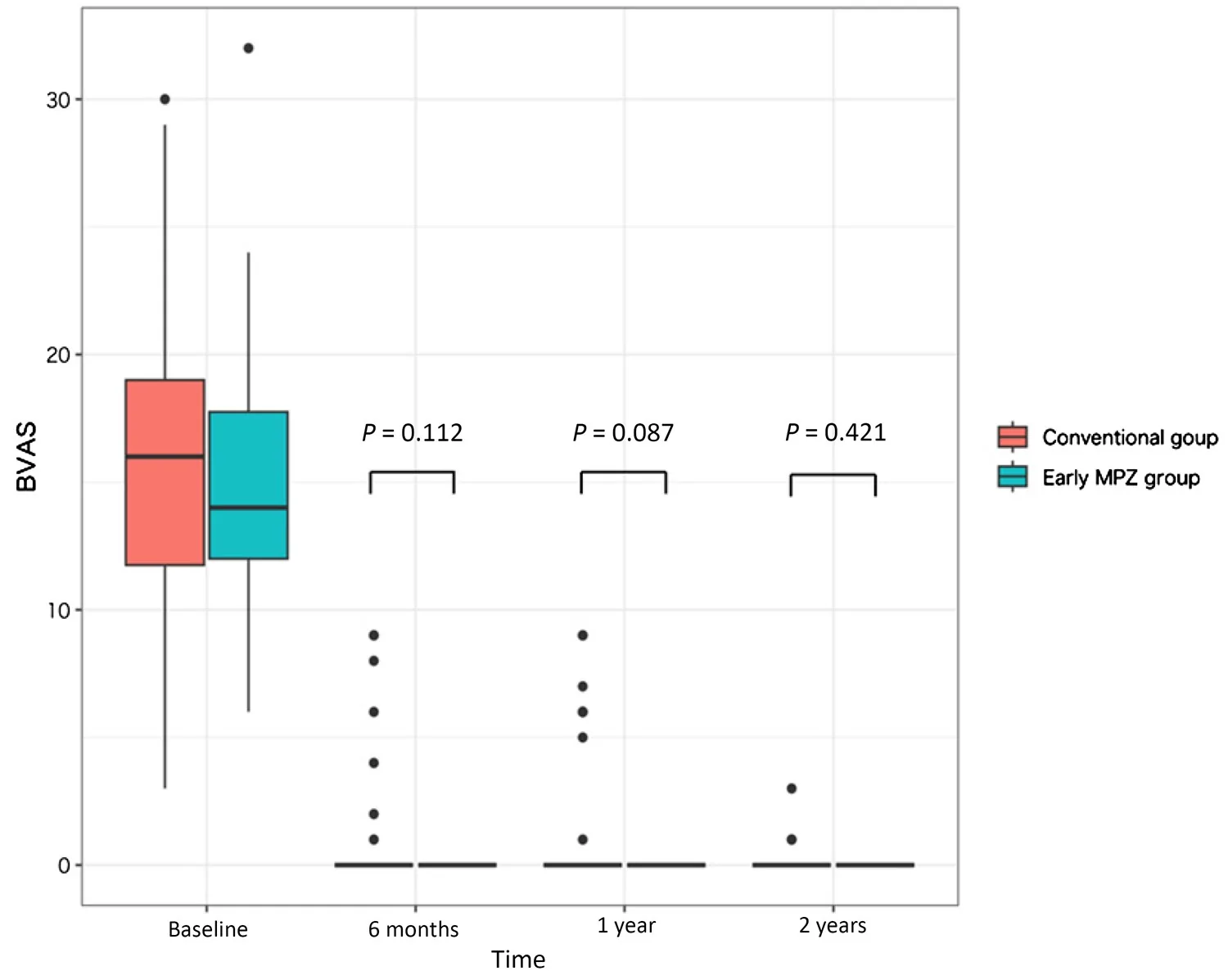
Longitudinal changes in disease activity (BVAS) in the conventional and early MPZ groups. Box-and-whisker plots showing BVAS at baseline, 6 months, 1 year and 2 years in the conventional remission induction group and the early MPZ initiation group. Boxes represent the interquartile range (IQR), the horizontal line indicates the median, and whiskers represent the minimum and maximum values excluding outliers. Dots indicate individual outliers. Between-group comparisons at each time point were performed using the Wilcoxon rank-sum test. No statistically significant differences were observed between groups at any time point. Abbreviation: MPZ: mepolizumab

### Glucocorticoid reduction

Prednisolone doses were lower in the early mepolizumab group at 6 months, 1 year, and 2 years (Fig. 2). The proportion of patients receiving prednisolone at a dose of ≤4 mg/day was 31% (5/16) in the early mepolizumab group and was 10% (5/48) in the conventional group at 6 months (*P = *0.104). At 1 year, the corresponding proportions were 69% (11/16) and 27% (13/48), respectively (*P = *0.006). At 2 years, among patients with available data, all 10 patients (100%) in the early mepolizumab group and 15 of 45 patients (33%) in the conventional group were receiving prednisolone at a dose of ≤4 mg/day (*P < *0.001). Complete GC discontinuation was achieved in 2 patients (13%) in the early mepolizumab group and in 2 patients (4%) in the conventional group at 1 year (*P = *0.258). At 2 years, GCs had been discontinued in 4 of 10 patients (40%) in the early mepolizumab group and in 4 of 45 patients (9%) in the conventional group (*P = *0.029) (Fig. 3).

**Figure 2:**
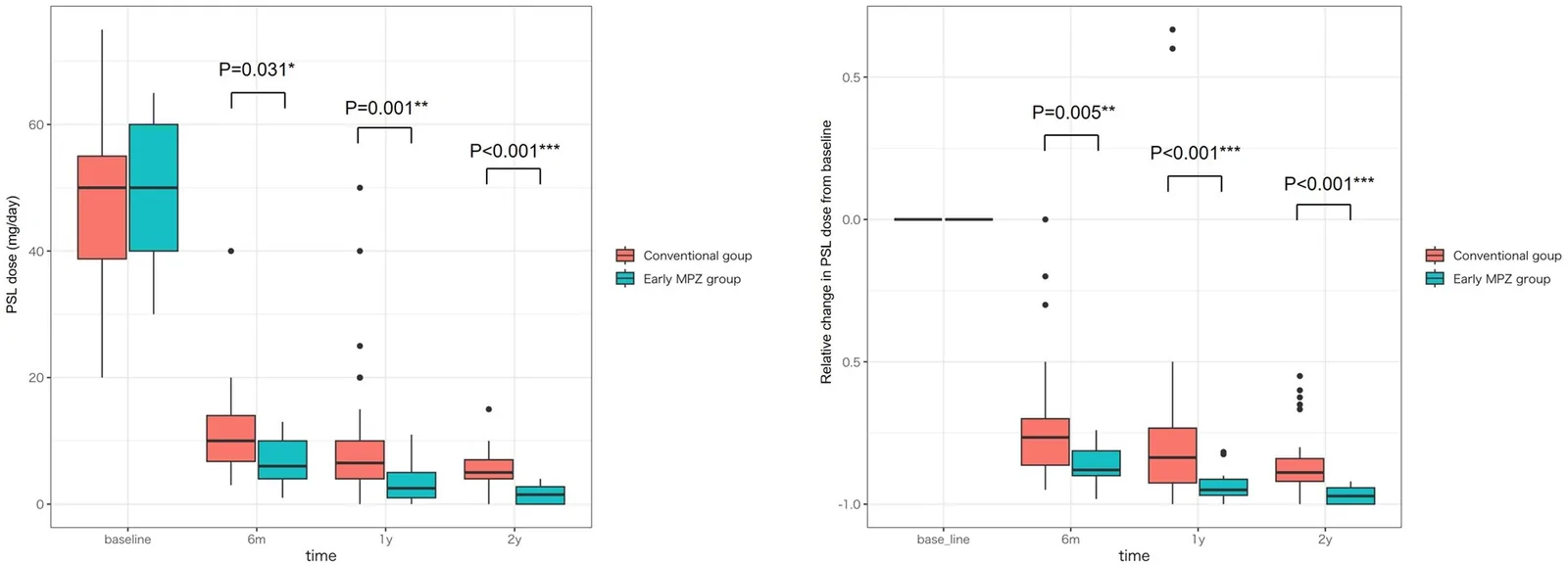
Changes in prednisolone dose and relative change from baseline over time. (A) Absolute prednisolone (PSL) dose at baseline, 6 months, 1 year, and 2 years in the conventional and early MPZ groups. (B) Relative change in PSL dose from baseline at 6 months, 1 year, and 2 years, calculated as (PSL dose at each time point / maximum PSL dose during remission induction) − 1. Box-and-whisker plots are presented as median with IQR. Between-group comparisons were performed using the Wilcoxon rank-sum test. The Early MPZ group showed lower PSL doses and greater relative reductions in PSL dose during follow-up. *P < 0.05, **P < 0.01, ***P < 0.001. Abbreviations: PSL: prednisolone; MPZ: mepolizumab

**Figure 3:**
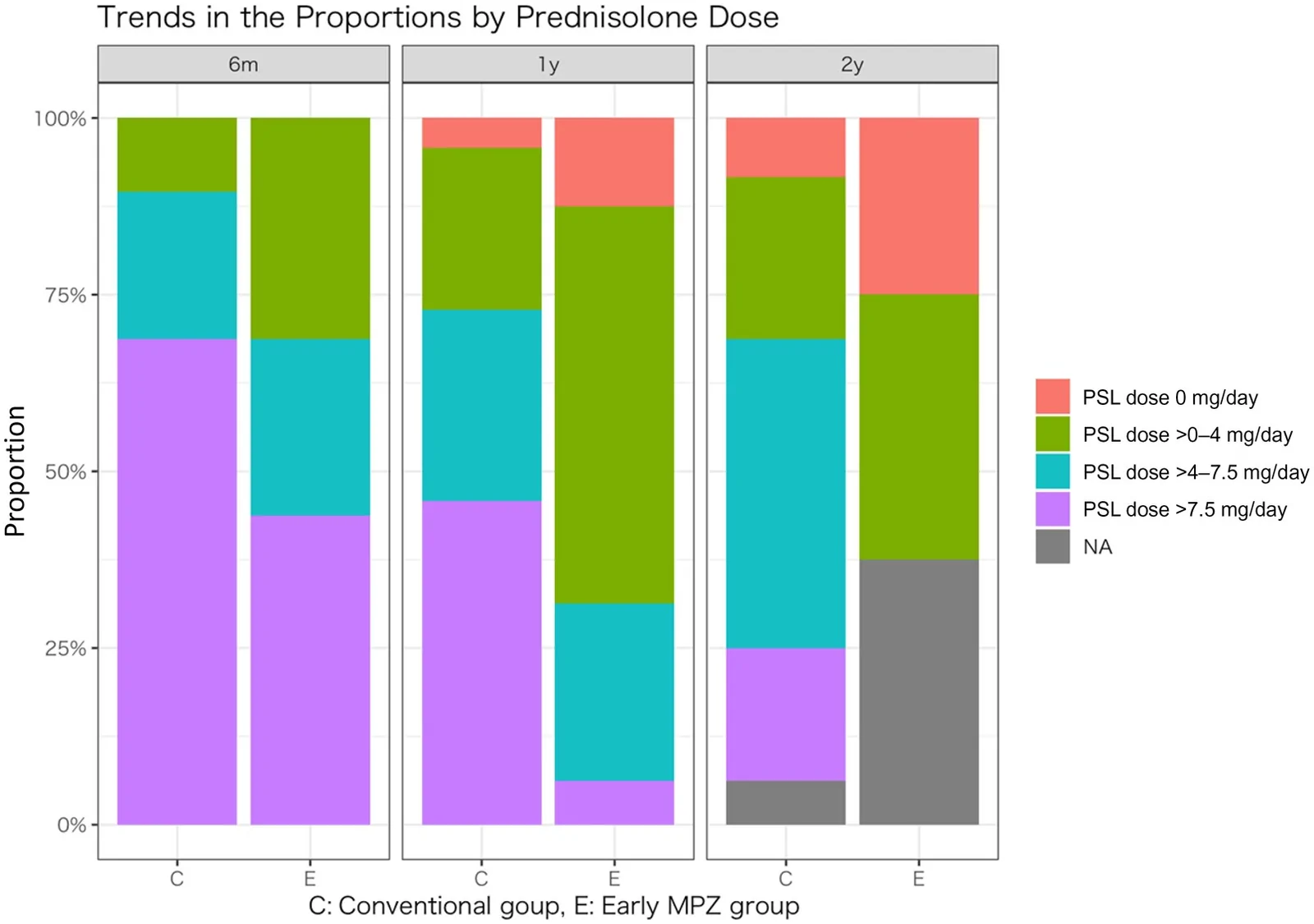
Proportions of patients according to prednisolone dose categories over time. Stacked bar plots showing the distribution of prednisolone dose categories (0 mg/day, >0–4 mg/day, >4–7.5 mg/day and >7.5 mg/day) at 6 months, 1 year and 2 years in the conventional remission induction group and the early MPZ initiation group. Percentages were calculated based on the available cases at each time point. The early MPZ group showed a progressive shift towards lower prednisolone dose categories over time. Abbreviations: PSL: prednisolone; MPZ: mepolizumab; NA: not available

### Treatment outcomes

Clinical remission, defined as BVAS = 0 and prednisolone ≤4 mg/day at the predefined assessment time points, was more frequent in the early mepolizumab group than in the conventional group.

At 6 months, clinical remission was achieved in 5 patients (31%) in the early mepolizumab group and in 5 patients (10%) in the conventional group (*P = *0.104). At 1 year (the primary end point), clinical remission was significantly more frequent in the early mepolizumab group (11/16, 69%) than in the conventional group (13/48, 27%) (*P = *0.006). At 2 years, clinical remission was observed in all evaluable patients in the early mepolizumab group (10/10, 100%), compared with 13 of 45 patients (29%) in the conventional group (*P < *0.001).

Within the first year after remission induction, relapse occurred in 5 patients (10%) in the conventional group, whereas no relapses were observed in the early mepolizumab group (*P = *0.320). During the first year, adverse events occurred in 1 of 16 patients (6%) in the early mepolizumab group and in 11 of 48 patients (23%) in the conventional group (*P = *0.266), while severe infections occurred in 1 of 16 patients (6%) and in 5 of 48 patients (10%), respectively (*P = *1.000). Between years 1 and 2, adverse events occurred in 1 of 10 patients (10%) and in 6 of 45 patients (13%), and severe infections occurred in 0 of 10 patients (0%) and in 3 of 45 patients (7%), respectively; none of these between-group differences was statistically significant. One patient in the conventional group died during follow-up (acute myocardial infarction), whereas no deaths occurred in the early mepolizumab group.

### Organ damage burden

Longitudinal changes in VDI during the first 2 years were assessed using a linear mixed-effects model with a patient-specific random intercept. Compared with the 6-month reference time point, VDI did not increase significantly at 1 year (β = 0.08, *P = *0.193) but was significantly higher at 2 years (β = 0.21, *P < *0.001). Across the follow-up time points, early mepolizumab initiation was associated with lower longitudinal VDI values than conventional treatment (β = −0.36, 95% CI −0.696 to −0.032; *P = *0.032). The estimated marginal mean VDI, averaged across the three assessment time points, was 0.92 (95% CI 0.63–1.21) in the early mepolizumab group and 1.28 (95% CI 1.12–1.45) in the conventional group (Fig. 4).

**Figure 4:**
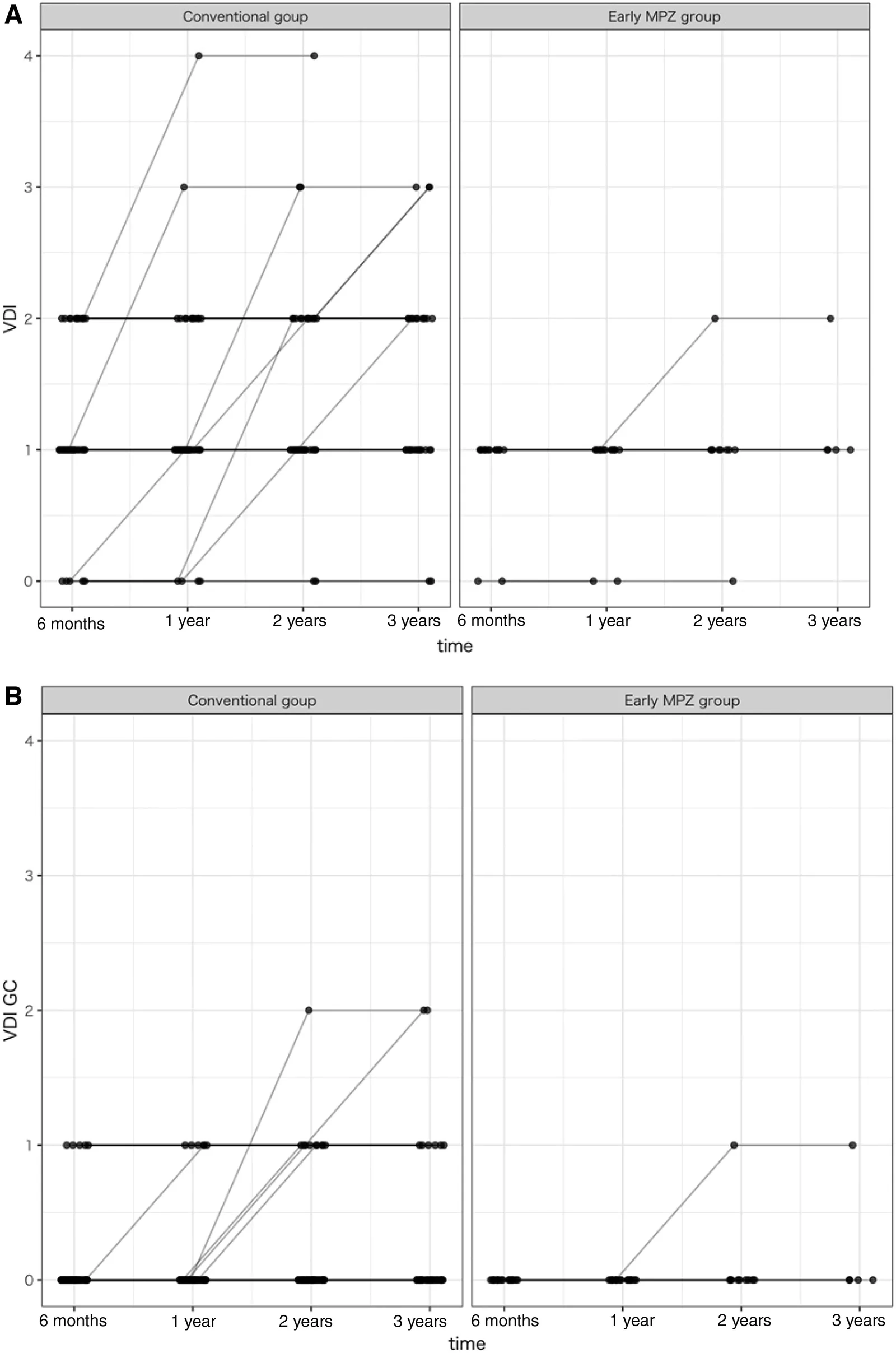
Longitudinal changes in organ damage assessed by the Vasculitis Damage Index. (A) Individual trajectories of total Vasculitis Damage Index (VDI) scores from 6 months to 3 years in the Conventional remission induction group and the early MPZ initiation group. (B) Glucocorticoid-related VDI items over time in both groups. Each dot represents an individual patient, and lines connect repeated measurements within the same patient. VDI scores remained generally lower in the early MPZ group during follow-up. Three-year values are shown descriptively and were not included in the mixed-effects analysis, which was restricted to the 2-year analysis window. Abbreviations: VDI: Vasculitis Damage Index; GC: glucocorticoid; MPZ: mepolizumab

Peripheral neuropathy remained common as irreversible damage in both groups (Supplementary Table S3). One early-mepolizumab initiation group patient developed avascular necrosis by 2 years, resulting in a 1-point VDI increase.

## Discussion

In this multicentre cohort study, initiation of mepolizumab within 3 months of the start of remission induction was associated with higher clinical remission rates at 1 and 2 years, greater GC reduction, more frequent GC discontinuation at 2 years, and lower longitudinal VDI values than conventional remission-induction therapy. Conventional immunosuppressive agents were also used less frequently in the early mepolizumab group. These findings suggest that early IL-5 inhibition may be a useful component of remission-induction strategies in selected patients with EGPA, although the observational design precludes causal inference.

MIRRA established the efficacy of mepolizumab in relapsing or refractory EGPA, and extension and real-world studies have demonstrated sustained GC-sparing effects [8–10, 29]. In the MIRRA open-label extension, 43% of patients achieved prednisolone ≤4 mg/day and 28% discontinued GC treatment [9]. In a European multicentre study, 58.3% of patients receiving mepolizumab 300 mg every 4 weeks achieved complete response and 41.7% discontinued GC treatment at 24 months [29]. In the MARS study, 68% received ≤4 mg/day GC and 36% were GC-free after long-term treatment [10]. In our study, all 10 early-group patients evaluable at 2 years received prednisolone ≤4 mg/day, and four discontinued GC treatment. Although direct comparisons are limited by differences in populations, definitions, and treatment duration, these findings suggest that earlier initiation may facilitate GC reduction.

A notable finding was the lower use of conventional immunosuppressive agents in the early mepolizumab group (Supplementary Table S1). This may indicate a reduced need for additional immunosuppression; however, physician-directed treatment, residual confounding by indication, and the older age of the early group may also explain this difference. It therefore cannot be attributed solely to early mepolizumab initiation.

Evidence regarding mepolizumab-containing remission-induction strategies remains limited. Ueno *et al.* reported outcomes comparable with CYC, with greater GC reduction [13], whereas Bettiol *et al.* described clinical improvement with sequential rituximab and mepolizumab [30]. However, mepolizumab was generally added for persistent respiratory disease, and most patients received 100 mg every 4 weeks. Mepolizumab should not be considered a replacement for CYC or rituximab in severe EGPA [31], but may represent an additional option in selected patients. Rather, our findings support further evaluation of early IL-5 inhibition as an additional component of remission-induction treatment in selected patients.

Organ damage accumulation is a major determinant of long-term prognosis in ANCA-associated vasculitis [25], reflecting both persistent disease activity and treatment-related toxicity, particularly GC exposure. VDI was not significantly higher at 1 year than at the 6-month reference point but increased by 2 years. Early mepolizumab initiation was associated with lower VDI values across the three assessment points. Because the mixed-effects model estimated the average group difference across time, this should be interpreted as a lower longitudinal VDI burden rather than a slower rate of damage accumulation or a causal protective effect.

Active BVAS domains largely resolved after remission induction, whereas peripheral neuropathy remained common as irreversible VDI damage in both groups (Supplementary Tables S2 and S3). This dissociation indicates that control of active vasculitis does not reverse established neurological damage and underscores the importance of treatment before irreversible nerve injury develops.

One early-group patient developed avascular necrosis by 2 years, indicating that GC-related damage may still occur despite subsequent GC reduction. Adverse events and severe infections were numerically less frequent, but event numbers were small and between-group differences were not significant; safety findings should therefore be interpreted descriptively.

Calendar-time bias was a concern because early mepolizumab initiation occurred predominantly in more recent years. In the sensitivity analysis restricted to patients who underwent remission induction in or after 2019, the direction of the VDI difference was consistent with the primary analysis, although it was not significant, and observation periods were similar between groups (Supplementary Table S4 and Supplementary Fig. S1). Thus, calendar-time differences alone may not fully account for the association, although residual calendar-time confounding and the limited sample preclude firm conclusions.

This study has several limitations. First, its retrospective observational design was subject to treatment-selection bias and residual confounding, and patient-level reasons for early or delayed mepolizumab initiation were not systematically recorded. Patients in the early mepolizumab group were older at diagnosis, and unmeasured factors, including physician preference, disease phenotype, eosinophil burden, comorbidities, and treatment tolerability, may have influenced treatment selection and outcomes. Eosinophil counts and the Five-Factor Score were not uniformly available. Second, the modest sample size, particularly in the early mepolizumab group (*n* = 16), limited statistical precision and precluded comprehensive multivariable or propensity score adjustment. Only 10 patients in this group had evaluable data at 2 years, and bias arising from differential or non-random missingness cannot be excluded. Third, GC tapering and conventional immunosuppressant use were not standardized across participating institutions. Because prednisolone dose was included in the definition of clinical remission, physician-directed tapering may have influenced remission outcomes. Fourth, nine patients in the conventional group subsequently initiated mepolizumab, potentially attenuating later between-group differences. The 3-month exposure threshold was operational and not prospectively validated. Finally, sparse VDI components and few safety events limited further analyses. Generalizability may be limited to Japanese patients treated at specialist centres using the approved 300 mg EGPA regimen. These findings should therefore be considered hypothesis-generating.

Despite these limitations, this multicentre cohort study provides clinically relevant real-world evidence that early mepolizumab initiation was associated with favourable clinical outcomes and a lower organ damage burden during the first 2 years of EGPA treatment. Prospective studies with standardized treatment and tapering protocols are needed to define the optimal timing, patient selection, and the role of mepolizumab in relation to conventional remission-induction therapy.

## Conclusion

In conclusion, initiation of mepolizumab within 3 months of the start of remission induction was associated with higher clinical remission rates at 1 and 2 years, reduced GC exposure, and a lower longitudinal organ damage burden during the first 2 years in patients with EGPA. Given the retrospective observational nature of this study, these findings should be interpreted as an association rather than a causal effect. Further prospective studies are required to determine the optimal timing and clinical indications for early IL-5 inhibition in EGPA.

This study was conducted in accordance with the Declaration of Helsinki. The study protocol was approved by the Institutional Review Board of Saga University Hospital (approval no. 2014-12-08), the representative institution of KVAS, with additional local approval obtained at participating institutions where required. Given the retrospective design, the requirement for written informed consent was waived, and information about the study was publicly disclosed to provide patients with the opportunity to opt out of participation.

## Supplementary Material

keag523_Supplementary_Data

## Data Availability

The data underlying this article are not publicly available due to ethical and privacy restrictions concerning individual patient data. The data may be made available from the corresponding author upon reasonable request, subject to approval by the relevant ethics committees and participating institutions.
