# Supplementary material for: Early mepolizumab initiation during remission induction is associated with lower organ damage burden in EGPA: a multicentre cohort study
Source: Rheumatology (Oxford). 2026 Sep 21;65(10):keag523. doi: 10.1093/rheumatology/keag523 (PMC13653373; doi:10.1093/rheumatology/keag523)
Supplement: Supplementary file 1 [file keag523_supplementary_data.zip › rhe-26-1119-File004.docx]

**Supplementary Table S1. Distribution and sequence of conventional immunosuppressive agents within 6 months after remission induction.**

| Line of therapy | Drug | Early MPZ group  (n=16) | | Conventional group  (n=48) | |
| --- | --- | --- | --- | --- | --- |
| **First-line**, n (%) | None | 10 | (62.5) | 13 | (27.1) |
|  | IVCY | 5 | (31.3) | 13 | (27.1) |
|  | POCY | 0 | (0.0) | 10 | (20.8) |
|  | RTX | 1 | (6.3) | 0 | (0.0) |
|  | MTX | 0 | (0.0) | 9 | (18.8) |
|  | AZP | 0 | (0.0) | 3 | (6.3) |
| **Second-line**, n (%) | None | 16 | (100.0) | 36 | (75.0) |
|  | POCY | 0 | (0.0) | 1 | (2.1) |
|  | AZP | 0 | (0.0) | 10 | (20.8) |
|  | TAC | 0 | (0.0) | 1 | (2.1) |
| **Third-line**, n (%) | None | 16 | (100.0) | 47 | (97.9) |
|  | MTX | 0 | (0.0) | 1 | (2.1) |
| **Any immunosuppressant use**, n (%) | | 6 | (37.5) | 35 | (72.9) |

Immunosuppressive therapy was assessed at 6 months after remission induction. First-, second-, and third-line therapies represent sequentially administered immunosuppressive agents within the first 6 months and do not necessarily indicate simultaneous use. “None” indicates that no additional immunosuppressive agents other than glucocorticoids were used at the respective line. Because these data are presented at the aggregate level, patient-level treatment sequences cannot be fully reconstructed and should be interpreted descriptively. Mepolizumab was not included in this table. Abbreviations: IVCY, intravenous cyclophosphamide; POCY, oral cyclophosphamide; RTX, rituximab; MTX, methotrexate; AZP, azathioprine; TAC, tacrolimus.

**Supplementary Table S2: BVAS domain involvement at baseline, 6 months, 1 year, and 2 years in the Early MPZ and Conventional groups**

|  | Baseline | | | 6 months | | | 1 year | | | 2 years | | |
| --- | --- | --- | --- | --- | --- | --- | --- | --- | --- | --- | --- | --- |
| BVAS domain | Early MPZ | Conventional | P value | Early MPZ | Conventional | P value | Early MPZ | Conventional | P value | Early MPZ | Conventional | P value |
| Systemic | 10/16 (63%) | 32/48 (67%) | 0.769 | 0/16 (0%) | 2/48 (4%) | 1.000 | 0/16 (0%) | 4/48 (8%) | 0.564 | 0/10 (0%) | 2/45 (4%) | 1.000 |
| Neurological | 15/16 (94%) | 41/48 (85%) | 0.667 | 0/16 (0%) | 5/48 (10%) | 0.319 | 0/16 (0%) | 6/48 (12%) | 0.323 | 0/10 (0%) | 0/45 (0%) | 1.000 |
| Sensory peripheral neuropathy | 15/16 (94%) | 41/48 (85%) | 0.667 | 0/16 (0%) | 4/48 (8%) | 0.564 | 0/16 (0%) | 5/48 (10%) | 0.319 | 0/10 (0%) | 0/45 (0%) | 1.000 |
| Motor mononeuritis multiplex | 9/16 (56%) | 25/48 (52%) | 1.000 | 0/16 (0%) | 2/48 (4%) | 1.000 | 0/16 (0%) | 2/48 (4%) | 1.000 | 0/10 (0%) | 0/45 (0%) | 1.000 |
| Skin | 8/16 (50%) | 24/48 (50%) | 1.000 | 0/16 (0%) | 1/48 (2%) | 1.000 | 0/16 (0%) | 3/48 (6%) | 0.567 | 0/10 (0%) | 0/45 (0%) | 1.000 |
| Ophthalmic/otologic/rhinologic | 10/16 (63%) | 29/48 (60%) | 1.000 | 0/16 (0%) | 2/48 (4%) | 1.000 | 0/16 (0%) | 2/48 (4%) | 1.000 | 0/10 (0%) | 1/45 (2%) | 1.000 |
| Pulmonary | 6/16 (38%) | 22/48 (46%) | 0.772 | 0/16 (0%) | 1/48 (2%) | 1.000 | 0/16 (0%) | 3/48 (6%) | 0.567 | 0/10 (0%) | 0/45 (0%) | 1.000 |
| Cardiac | 6/16 (38%) | 8/48 (17%) | 0.095 | 0/16 (0%) | 0/48 (0%) | 1.000 | 0/16 (0%) | 0/48 (0%) | 1.000 | 0/10 (0%) | 0/45 (0%) | 1.000 |
| Renal | 2/16 (13%) | 10/48 (21%) | 0.714 | 0/16 (0%) | 1/48 (2%) | 1.000 | 0/16 (0%) | 0/48 (0%) | 1.000 | 0/10 (0%) | 0/45 (0%) | 1.000 |
| Gastrointestinal | 0/16 (0%) | 3/48 (6%) | 0.567 | 0/16 (0%) | 0/48 (0%) | 1.000 | 0/16 (0%) | 0/48 (0%) | 1.000 | 0/10 (0%) | 0/45 (0%) | 1.000 |

Data are presented as the number of patients with involvement in each Birmingham Vasculitis Activity Score (BVAS) domain divided by the number of evaluable patients at each assessment time point, with percentages in parentheses. Domain involvement was defined as the presence of at least one active BVAS item within the corresponding domain. Sensory peripheral neuropathy and motor mononeuritis multiplex are shown as selected components of the neurological domain and are not mutually exclusive. At baseline, 6 months, and 1 year, 16 patients in the Early MPZ group and 48 patients in the Conventional group were evaluable. At 2 years, data were available for 10 and 45 patients, respectively. Between-group comparisons were performed using two-sided Fisher’s exact tests. Because of the small number of residual events and multiple domain-level comparisons, the results should be interpreted as descriptive and exploratory; no adjustment for multiple testing was performed.

Abbreviations: BVAS, Birmingham Vasculitis Activity Score; MPZ, mepolizumab.

**Supplementary Table S3. Item-level distribution of Vasculitis Damage Index components at 6 months, 1 year, and 2 years in the Early MPZ and Conventional groups**

| VDI item | Time point | Early MPZ group | Conventional group |
| --- | --- | --- | --- |
| Severe muscle atrophy or muscle weakness | 6 mo | 0 | 2 |
|  | 1 yr | 0 | 2 |
|  | 2 yr | 0 | 2/45 |
| Osteoporosis with fracture or vertebral collapse | 6 mo | 0 | 1 |
|  | 1 yr | 0 | 1 |
|  | 2 yr | 0 | 2/45 |
| Avascular necrosis | 6 mo | - | - |
|  | 1 yr | 0 | 2 |
|  | 2 yr | 1/10 | 2/45 |
| Cataract | 6 mo | 0 | 1 |
|  | 1 yr | 0 | 2 |
|  | 2 yr | 0 | 4/45 |
| Diabetes mellitus | 6 mo | 0 | 1 |
|  | 1 yr | 0 | 1 |
|  | 2 yr | 0 | 1/45 |
| Hearing loss | 6 mo | 0 | 0 |
|  | 1 yr | 0 | 1 |
|  | 2 yr | 0 | 1/45 |
| Nasal obstruction, chronic nasal discharge, or nasal crusting | 6 mo | 0 | 1 |
|  | 1 yr | 0 | 1 |
|  | 2 yr | 0 | 1/45 |
| Chronic sinusitis | 6 mo | 0 | 2 |
|  | 1 yr | 0 | 3 |
|  | 2 yr | 0 | 3/45 |
| Pulmonary fibrosis | 6 mo | 0 | 0 |
|  | 1 yr | 0 | 1 |
|  | 2 yr | 0 | 1/45 |
| Chronic asthma | 6 mo | 0 | 3 |
|  | 1 yr | 0 | 3 |
|  | 2 yr | 0 | 3/45 |
| Angina, angioplasty, or coronary artery bypass surgery | 6 mo | 0 | 1 |
|  | 1 yr | 0 | 1 |
|  | 2 yr | 0 | 1/45 |
| Myocardial infarction | 6 mo | 0 | 0 |
|  | 1 yr | 0 | 0 |
|  | 2 yr | 0 | 0 |
| Cardiomyopathy | 6 mo | 0 | 1 |
|  | 1 yr | 0 | 1 |
|  | 2 yr | 0 | 1/45 |
| Diastolic blood pressure >95 mmHg | 6 mo | 1 | 1 |
|  | 1 yr | 1 | 1 |
|  | 2 yr | 1/10 | 1/45 |
| End-stage renal disease | 6 mo | 0 | 0 |
|  | 1 yr | 0 | 0 |
|  | 2 yr | 0 | 0 |
| Cerebrovascular accident | 6 mo | 0 | 2 |
|  | 1 yr | 0 | 2 |
|  | 2 yr | 0 | 1/45 |
| Cranial neuropathy | 6 mo | 0 | 0 |
|  | 1 yr | 0 | 0 |
|  | 2 yr | 0 | 0 |
| Peripheral neuropathy | 6 mo | 14 | 38 |
|  | 1 yr | 14 | 38 |
|  | 2 yr | 9/10 | 36/45 |
| Visual impairment or diplopia | 6 mo | 0 | 1 |
|  | 1 yr | 0 | 1 |
|  | 2 yr | 0 | 1/45 |
| Malignancy | 6 mo | 0 | 0 |
|  | 1 yr | 0 | 0 |
|  | 2 yr | 0 | 0 |

Data are shown as the number of patients with each Vasculitis Damage Index item at each time point. Because follow-up duration varied across patients, the number of patients with each item may decrease at later time points. Because of the small sample size and sparse events, formal statistical comparisons for individual items were not emphasized, and these findings should be interpreted as exploratory.

**Supplementary Table S4. Baseline characteristics of patients who underwent remission induction in or after 2019.**

|  | All patients  (N=35) | | Early MPZ group  (N=16) | | Conventional group  (N=19) | | p value |
| --- | --- | --- | --- | --- | --- | --- | --- |
| Male:Female (Female%) | 15:20 | (57.1) | 6:10 | (62.5) | 9:10 | (52.6) | 0.734 |
| Median age at diagnosis (range) | 62 | (29-88) | 67 | (31-88) | 58 | (29-84) | 0.059 |
| Observation period, months (range) | 25.5 | (12.0-72.5) | 24.9 | (12.3-37.5) | 26.7 | (12.0-72.5) | 0.320 |
| Bronchial asthma, n (%) | 21 | (60.0) | 11 | (68.8) | 10 | (52.6) | 0.491 |
| MPO-ANCA, n (%) | 13 | (37.1) | 7 | (43.8) | 6 | (31.6) | 0.503 |
| PR3-ANCA, n (%) | 0 | (0.0) | 0 | (0.0) | 0 | (0.0) | - |
| Median initial BVAS (range) | 14 | (3-32) | 14 | (6-32) | 14 | (3-21) | 0.425 |
| BVAS clinical symptoms (%) |  |  |  |  |  |  |  |
| Systemic symptoms (%) | 20 | (57.1) | 10 | (62.5) | 10 | (52.6) | 0.734 |
| Neurological (%) | 30 | (85.7) | 15 | (93.8) | 15 | (78.9) | 0.347 |
| Skin (%) | 15 | (42.9) | 8 | (50.0) | 7 | (36.9) | 0.506 |
| Ophthalmic/Otologic/Rhinologic (%) | 24 | (68.6) | 10 | (62.5) | 14 | (73.7) | 0.716 |
| Pulmonary (%) | 14 | (40.0) | 6 | (37.5) | 8 | (42.2) | 1.000 |
| Cardiac (%) | 11 | (31.4) | 6 | (37.5) | 5 | (26.3) | 0.716 |
| Renal (%) | 4 | (11.4) | 2 | (12.5) | 2 | (10.5) | 1.000 |
| Gastrointestinal (%) | 1 | (2.9) | 0 | (0.0) | 1 | (5.3) | 1.000 |
| Treatment |  |  |  |  |  |  |  |
| Median Maximum PSL Dose, mg (Range) | 50 | (20-65) | 50 | (30-65) | 50 | (20-65) | 0.501 |
| Initial GC Pulse, n (%) | 18 | (51.4) | 7 | (43.8) | 11 | (57.9) | 0.505 |
| Median PSL after 6 months, mg (range) | 7 | (1-15) | 6 | (1-13) | 7 | (3-15) | 0.280 |
| Median PSL after 1 year, mg (range) | 4 | (0-15) | 2.5 | (0-11) | 5 | (0-15) | 0.114 |
| Median PSL after 2 years, mg (range) | 3 | (0-9) | 1.5 | (0-4) | 5 | (0-9) | 0.039^*^ |
| Immunosuppressant combination within 6 months, n (%) | 24 | (68.6) | 6 | (37.5) | 18 | (94.7) | <0.001^***^ |
| Immunosuppressant combination after 1 year, n (%) | 10 | (28.6) | 2 | (12.5) | 8 | (42.1) | 0.071 |
| Immunosuppressant combination after 2 years, n (%) | 11/26 | (42.3) | 1/10 | (10.0) | 10/16 | (62.5) | 0.014^*^ |
| Prognosis |  |  |  |  |  |  |  |
| BVAS=0 achieved, n (%) | 34 | (97.1) | 16 | (100.0) | 18 | (94.7) | 1.000 |
| Clinical remission achieved at 6 months, n (%) | 9 | (25.7) | 5 | (31.3) | 4 | (21.1) | 0.700 |
| Clinical remission achieved at 1 year, n (%) | 20 | (57.1) | 11 | (68.8) | 9 | (47.4) | 0.306 |
| Clinical remission achieved at 2 years, n (%) | 17/26 | (65.4) | 10/10 | (100.0) | 7/16 | (43.8) | 0.004^**^ |
| Ideal remission at 1 year, n (%) | 4 | (11.4) | 2 | (12.5) | 2 | (10.5) | 1.000 |
| Ideal remission at 2 years, n (%) | 8/26 | (30.8) | 4/10 | (40.0) | 4/16 | (25.0) | 0.664 |
| Relapse within 1 year, n (%) | 3 | (8.6) | 0 | (0.0) | 3 | (15.8) | 0.234 |
| Relapse within 1–2 years, n (%) | 0 | (0.0) | 0 | (0.0) | 0 | (0.0) | - |
| Adverse events within 1 year, n (%) | 1 | (2.9) | 1 | (6.3) | 0 | (0.0) | 0.457 |
| Adverse events within 1-2 years, n (%) | 3/26 | (11.5) | 1/10 | (10.0) | 2/16 | (12.5) | 1.000 |
| Severe infections within 1 year, n (%) | 1 | (2.9) | 1 | (6.3) | 0 | (0.0) | 0.457 |
| Severe infections within 1-2 years, n (%) | 1/26 | (3.9) | 0 | (0.0) | 1/16 | (6.3) | 1.000 |
| Death during follow up, n (%) | 0 | (0.0) | 0 | (0.0) | 0 | (0.0) | - |

Baseline demographic, clinical, and laboratory characteristics of patients who underwent remission induction in or after 2019 in the Conventional remission induction group and the Early MPZ initiation group. Continuous variables are expressed as median (range), and categorical variables as number (percentage). Statistical comparisons were performed as described for Table 1.

Abbreviations are the same as in Table 1. *P<0.05, **P<0.01, ***P<0.001.

**Supplementary Figure S1. Longitudinal changes in organ damage assessed by the Vasculitis Damage Index (VDI) among patients who underwent remission induction in or after 2019.**

Individual trajectories of total VDI scores from 6 months to 3 years in the Conventional remission induction group and the Early MPZ initiation group, restricted to patients who underwent remission induction in or after 2019. Each dot represents an individual patient, and lines connect repeated measurements within the same patient.

VDI values remained numerically lower in the Early MPZ group throughout follow-up, consistent with the primary analysis.
